# Psychodermatology—A special edition of Skin Health and Disease

**DOI:** 10.1002/ski2.192

**Published:** 2022-11-20

**Authors:** Ewan A. Langan, George W. M. Millington

**Affiliations:** ^1^ Department of Dermatology University of Lübeck Lübeck Germany; ^2^ Dermatological Sciences University of Manchester Manchester UK; ^3^ Dermatology Department Norfolk and Norwich University Hospital Norwich UK; ^4^ Norwich Medical School University of East Anglia Norwich UK

1

Psychodermatology is the field dedicated to the study and treatment of patients with primary skin diseases which are associated with psychosocial morbidity, for example, psoriasis, atopic dermatitis and skin cancer, as well as psychiatric disorders which present to dermatologists, including trichotillomania and delusional parasitosis.^1^

Given that both the skin and nervous system arise from the same embryological source, ectoderm, their intimate connection is hard‐wired before birth. Whilst neurocutaneous genodermatoses^2^ such as neurofibromatosis and tuberous sclerosis are perhaps the best proof of the neuro‐cutaneous circuit, they only represent the tip of the brain‐skin axis iceberg. Indeed, a plethora of neuropeptides are actually produced in the skin, playing a role in itch and skin inflammation. The hair follicle even has a peripheral equivalent of the hypothalamic‐pituitary‐adrenal axis.^3^, ^4^, ^5^

In this special edition of *Skin Health and Disease* we aim to showcase the breadth and depth of psychodermatology research, covering genetic and acquired skin diseases, involving patients across the entire age spectrum suffering from primary skin or psychiatric diagnoses.^6^, ^7^, ^8^, ^9^, ^10^, ^11^, ^12^, ^13^, ^14^, ^15^, ^16^, ^17^, ^18^, ^19^ Furthermore, we report the potential of medications to trigger autoimmune blistering disease in patients with neuropsychological disorders.^6^ The issue also includes robust prospective epidemiological data from national registries examining associations between mental health and psoriatic arthritis in patients with psoriasis as well as charting the development of psychodermatology clinics and liaison services^7^ and the establishment of patient reported outcomes measures for body image in dermatology is detailed.^8^ Finally, Frewen *et al*. show how psychodermatology can even get under the skin of healthcare professionals.^9^

Specialised, multi‐disciplinary clinics seem best placed to meet the growing need for psychodermatological service provision. However, despite improvements in the delivery of psychodermatology services in the UK, a recent assessment concluded that overall provision remains poor.^1^

Any attempts to improve the provision of psychodermatology treatment services will likely require firm epidemiological evidence of clinic need, basic science research to identify treatment strategies and targets, a critical evaluation of the efficacy of existing services, cost‐effective and evidence‐based treatment interventions, and the embracement of new technology to support service delivery. After centuries of Cartesian dualism, an integrated understanding of the nuts and bolts of our neurocutaneous circuitry is long overdue.

## CONFLICT OF INTEREST

Ewan A. Langan is Deputy Editor in Chief of SHD; George Millington is Editor in Chief of SHD.

## FUNDING INFORMATION

This article received no specific grant from any funding agency in the public, commercial, or not‐for profit sectors.

## AUTHOR CONTRIBUTIONS

**Ewan A. Langan**: Writing – original draft (Lead); Writing – review & editing (Lead). **George W. M. Millington**: Writing – original draft (Supporting); Writing – review & editing (Supporting).

## ETHICAL STATEMENT

Not applicable.

## Data Availability

Data sharing is not applicable to this article as no new data were created or analysed in this study.
